# Contributing effects of sarcopenia on cancer occurrence: novel evidence based on NHANES 1999–2020 and two-sample mendelian randomization study

**DOI:** 10.1093/oncolo/oyaf369

**Published:** 2025-11-05

**Authors:** Zheng Xu, Xiaoru Luo, Wuliang Diao, Xinlinzi Tang, Yue Zhang, Jiahao Wang, Zichao Jiang, Ting Lei

**Affiliations:** The Seventh Clinical Medicial College of Guangzhou University of Chinese Medicine, Shenzhen, China; The Seventh Clinical Medicial College of Guangzhou University of Chinese Medicine, Shenzhen, China; Department of Plastic Surgery, Affiliated Hospital of Guizhou Medical University, Guiyang, China; The Seventh Clinical Medicial College of Guangzhou University of Chinese Medicine, Shenzhen, China; National Clinical Research Center for Geriatric Disorders, Xiangya Hospital, Central South University, Changsha, China; The School of Medicine, Nankai University, Tianjin, China; Department of Orthopedic Surgery, The First Affiliated Hospital, College of Medicine, Zhejiang University, Hangzhou, China; Department of Orthopedic Surgery, The First Affiliated Hospital, College of Medicine, Zhejiang University, Hangzhou, China

**Keywords:** sarcopenia, muscle, cancer, NHANES, Mendelian randomization

## Abstract

**Background:**

Sarcopenia is associated with worse prognosis in patients with cancer, and patients with cancer usually have poor muscle condition. However, it is still not clear whether sarcopenia contributes to cancer occurrence.

**Methods:**

This study utilized data from the National Health and Nutrition Examination Survey (NHANES) 1999–2020 to assess the relationship between sarcopenia and cancer. Key sarcopenia indicators, including lean mass measurements and sarcopenia diagnosed by European Working Group on Sarcopenia in Older People (EWGSOP) or Foundation for the National Institutes of Health (FNIH) criteria, were analyzed using quantile logistic regression models. A two-sample Mendelian Randomization (MR) approach was employed to explore the causal link between sarcopenia and cancer, leveraging genetic variants as instrumental variables.

**Results:**

We found that after adjusting covariates, left arm lean mass, left leg lean mass and appendicular lean mass (ALM) were found to have significant negative influences on the occurrence of overall-cancer types, particularly in individuals over 40 years old. Sarcopenia (FNIH) significantly increased the risk of overall-cancer types, especially colon, ovarian, and uterine cancers. Smooth curve fitting showed that cancer incidence decreased with increasing muscle mass. MR analysis confirmed that ALM and whole body lean mass (WBLM) had causally negative effects on cancer, while grip strength and sarcopenia (by EWGSOP/FNIH criteria) demonstrated significant causal effects on specific cancer types, including lymphoid hematopoietic cancer, lymphoid leukemia, and breast cancer.

**Conclusion:**

Sarcopenia significantly impacts the incidence of various cancers and may causally contribute to cancer development. Managing sarcopenia could potentially benefit cancer prevention and treatment strategies.

## Graphical Abstract

**Graphical Abstract:**
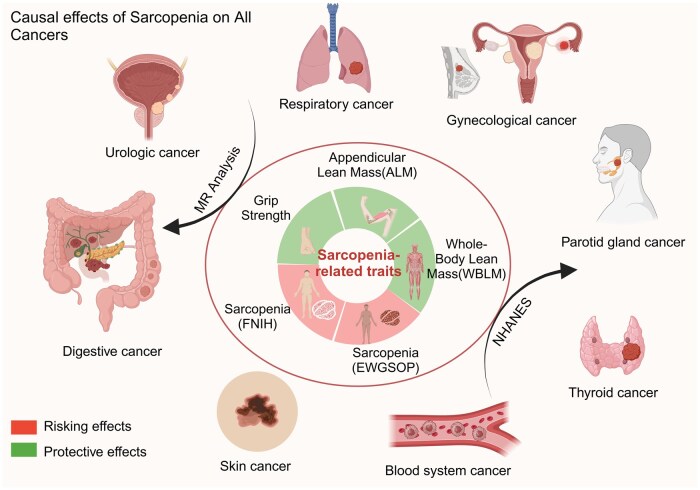

Implications for Practice This study emphasizes the vital importance of sarcopenia management in both oncology and primary care. Clinicians are encouraged to incorporate routine assessments of muscle mass and strength, using standardized criteria such as those established by the FNIH or the EWGSOP, for at-risk populations, particularly individuals over 40 years of age. The findings suggest that interventions aimed at preventing or reversing sarcopenia, including resistance training and nutritional support focused on increasing lean mass, could play a vital role in comprehensive cancer prevention strategies. Moreover, for patients diagnosed with specific cancers, including colon, ovarian, uterine and breast cancer, proactively addressing sarcopenia may not only enhance their overall health status but also potentially impact disease outcomes positively.

## Introduction

Cancer is one of the main reasons causing mortality and global disease burden. In 2019, it was estimated that cancer had 23.6 million new cases and caused 10 million deaths in the world.^1^ In 2020, cancer led to the second most deaths and years of life lost, only after cardiovascular diseases, and the cancer burden will continue to increase in the following 2 decades.^1^ Breast cancer and lung cancer caused the most deaths in women and men, respectively.^2^ Many risk factors have been identified to be related to cancer, such as cigarette smoking, exposure to ionizing radiation or UV radiation, alcohol drinking, occupation, diet habits and physical activity, and so on. Identifying and controlling the risk factors will contribute to lowering the incidence of cancer diseases.

Recently research found that sarcopenia was associated with worse survival and poor prognosis in patients with cancer. Sarcopenia is a syndrome characterized by progressive loss of muscle mass, loss of muscle strength and poor physical performance, which is related to impaired mobility, longer hospital stays, and higher mortality.^3^ Cancer disease population had a prevalence of sarcopenia as 35.3%,^4^ which was higher than that of normal old population, as 10-27% using different criteria.^5^ A cohort study reported that patients with cancer had a more decrease in gait speed than controls without cancers before cancer diagnosis, and a decline in appendicular lean mass (ALM) after diagnosis. Slow gait speed was related to the increased mortality and disability.^6^ In gynecologic cancers, including cervical, ovarian, uterine, vaginal, and vulvar cancer, sarcopenia was associated with worse overall survival and progression-free survival, with a pooled hazard ratio (HR) of 2.61 and 1.37, respectively.^7^ Every standard deviation decrease in lean mass could also cause increase of cancer mortality in different cancer types.^8^ However, although the association between sarcopenia and cancer exists, no current studies have elucidated whether sarcopenia results in cancer. Given that sarcopenia management possibly contributes to improving survival rate and decreasing mortality in patients with cancer, it is important to verify the causal relationship between sarcopenia and cancer.

National Health and Nutrition Examination Survey (NHANES) is an investigation conducted among American population. It records health relevant data from various aspects, including specific disease status, diet habits, physical examination results, laboratory examination results, medical conditions, and so on, to provide samples with high quality to explore the relationship between exposures and health outcomes. Therefore, NHANES can benefit the investigation of relationship between sarcopenia and cancer.^9^^,^^10^ In addition, Mendelian randomization (MR) is an effective epidemiological method to explore the causal relationship between exposures and outcomes from the genetic aspects. According to the rules of Mendel’s laws of inheritance, genetic variants are randomly allocated to offspring during the reproductive process. These genetic variants remain stable throughout an individual’s development and are not altered by environmental influences. Additionally, genetic variants can be accurately measured through methods such as gene sequencing. Therefore, it is possible to determine whether a causal relationship exists between exposures and outcomes by using genetic variants as instrumental variables and analyzing the variants related to both exposures and outcomes.^11^ MR analysis has been previously applied to investigate risking or protective factors of diseases and explore potential diagnosis markers or therapeutic targets. For example, one MR analysis investigated the association between serum 25(OH)D concentration and sarcopenia, and found that increased serum 25(OH)D concentration until 20 ng/mL rapidly decreased the risk of sarcopenia, which stressed the importance of serum 25(OH)D in sarcopenia prevention.^12^ This epidemiological method could be used to explore the causal association between sarcopenia and cancer, which has not been reported before.

In this study, we aim to conduct an observational study based on NHANES and MR analysis to explore whether there is a causal relationship between sarcopenia and cancer.

## Methods

### Study design

This study was composed of two parts. Firstly, we conducted an observational study based on participants’ data from NHANES to investigate the association between sarcopenia and cancers, during which the quantile logistic regression and the smooth curve fitting method were used. Secondly, we used an univariable two sample MR analysis to investigate the causal relationship between sarcopenia and cancers (cancer outcomes were obtained from 38 independent cancer-specific consortium datasets) from genetical aspects, based on summary statistics data acquired from the genome-wide association study (GWAS).

### The observational study based on NHANES

#### Data source and population information

The data were sourced from the National Health and Nutrition Examination Survey (NHANES) conducted from 1999 to 2020. The sarcopenia-related exposures included left arm lean mass, left leg lean mass, and appendicular lean mass (ALM). The study examined overall cancer incidence and various specific cancer types. Out of 124,822 total participants, 66,140 were excluded for lack of cancer information, and 32,771 were removed due to missing sarcopenia data. Ultimately, 25,911 participants were included in the final analysis. Table 1 presents the baseline information of participants categorized by sarcopenia status.

**Table 1.** Baseline data of participants in different groups grouped by sarcopenia.

SARCOPENIA FNIH	no	yes	P-value
**N**	22211	3700	
**AGE(years old)**	(22211) 44.037 ± 16.044	(3700) 54.419 ± 17.371	<0.001
**BMI VALUE(kg/m^2^)**	(22211) 28.098 ± 6.307	(3700) 32.209 ± 7.423	<0.001
**GENDER**			<0.001
**male**	11104 (49.993%)	1968 (53.189%)	
**female**	11107 (50.007%)	1732 (46.811%)	
**RACE.HISPANIC.ORIGIN**			<0.001
**Mexican American**	3676 (16.550%)	1332 (36.000%)	
**Other Hispanic**	1360 (6.123%)	307 (8.297%)	
**Non-Hispanic White**	10013 (45.081%)	1471 (39.757%)	
**Non-Hispanic Black**	5202 (23.421%)	329 (8.892%)	
**Other Race-Including MultiRacial**	1960 (8.824%)	261 (7.054%)	
**TOTAL ENERGY INTAKE(kcal)**	(21157) 2219.666 ± 1051.787	(3514) 1902.387 ± 913.826	<0.001
**SMOKING STATUS**			<0.001
**no**	12045 (54.279%)	1983 (53.624%)	
**past smoking**	4760 (21.450%)	1057 (28.583%)	
**current smoking**	5386 (24.271%)	658 (17.793%)	
**ALCOHOL DRINKING**			<0.001
**no**	5651 (27.401%)	1202 (34.770%)	
**yes**	14972 (72.599%)	2255 (65.230%)	
**LEFT ARM LEAN(g)**	(22102) 2989.255 ± 1040.255	(3601) 2734.523 ± 1021.083	<0.001
**LEFT LEG LEAN(g)**	(22202) 8204.739 ± 2209.119	(3351) 7151.145 ± 2130.545	<0.001
**APPENDICULAR LEAN MASS(kg)**	(22211) 22.674 ± 6.381	(3700) 18.452 ± 6.034	<0.001
**CANCER**			<0.001
**no**	20833 (93.796%)	3376 (91.243%)	
**yes**	1378 (6.204%)	324 (8.757%)	

Results: (N)Mean+SD/N(%). BMI: body mass index. p < 0.05 means significant statistical differences.

#### Sarcopenia and cancer status

According to the Foundation for the National Institutes of Health (FNIH), the value of appendicular lean mass adjusted by body mass index (BMI) was calculated to define sarcopenia, with the value smaller than 0.789 kg/m^2^ and 0.512 kg/m^2^ in sarcopenia male and female patients, respectively. The arm and leg lean mass were measured by Dual-energy x-ray absorptiometry (DXA). Appendicular lean mass was calculated as the sum of arms and legs lean mass. For the DXA examination, participants who were pregnant or had history of radiographic contrast material use or had weight over 450 pounds or height over 6’5’’ were excluded. Cancer participants were determined as those who were told by health professionals to have caner or a malignancy, with the cancer type information.

#### Covariates information

Covariates included age, gender, BMI, race, total energy intake, smoking status, and alcohol consumption. Participants were classified into two age groups: ≤40 years and >40 years. BMI was calculated as body weight (kg) divided by height squared (m^2^). Total energy intake was assessed using the USDA’s Food and Nutrient Database for Dietary Studies (FNDDS). Racial categories included Mexican American, Other Hispanic, Non-Hispanic White, and Other Race-Including Multi Racial. Smoking status was categorized as non-smoker, past smoker, or current smoker based on whether participants had smoked at least 100 cigarettes in their lifetime. Alcohol consumption was defined by having at least 12 alcoholic drinks in one year.

### Mendelian randomization

Exposure data included ALM, WBLM, grip strength, and sarcopenia diagnosed by EWGSOP or FNIH criteria. WBLM (454,850 cases) and grip strength data (461,026 cases) were acquired from the UK Biobank. ALM (PMID: 33097823, 450,243 cases), sarcopenia (EWGSOP) (PMID: 33510174, 48,596 cases), and sarcopenia (FNIH) (PMID: 33510174, 20,335 cases) data were obtained from the GWAS Catalog (https://www.ebi.ac.uk/gwas/home). GWAS data for all cancer (87,531 cases and 314,193 controls) and diverse cancers were sourced from the Finngen GWAS summary statistics (https://www.finngen.fi/en). Summary information of datasets is listed in **Supplemental Dataset 1**.

For genetic instrument selection and two-sample Mendelian randomization analysis, SNPs were selected based on three criteria to serve as IVs to investigate the relationship between sarcopenia-related traits and cancer. Firstly, SNPs influenced only the exposures (ALM, WBLM, grip strength, sarcopenia (EWGSOP), and sarcopenia (FNIH)). SNPs were extracted when p_exposure_<5e^−8^, and those with LD were excluded by setting r^2^=0.001 in a 10,000 kb LD window. F-statistics for SNPs were calculated, including those with F-statistics greater than 10. Secondly, SNPs influencing confounding factors were eliminated. Thirdly, SNPs not directly associated with cancer outcomes were selected by setting p_outcome_<5e^−5^. The SNPs included in the analysis are listed in **Supplemental Dataset 2-6**. For statistical analysis, the IVW method was mainly used to evaluate the relationship between sarcopenia and cancer. A fixed effect model was utilized when the number of SNPs was between 1 to 3, and a random effect model was used when SNPs were more than 3. The OR along with the 95% CI was used to describe the association, with p < 0.05 considered statistically significant. R software (Version: 4.2.2, https://www.r-project.org/) was used for all statistical analyses.

### Statistical analysis

Quantile logistic regression models were used to assess the relationship between sarcopenia, sarcopenia-related traits, and cancer occurrence. The Chi-square test was applied for classical data, while the Kruskal-Wallis test was used for quantitative data. Smooth curve fitting was employed to evaluate the relationship between sarcopenia-related traits and cancer incidence. A significant difference was defined as p < 0.05 for all statistical analyses. R software (Version: 4.2.2, https://www.r-project.org/) was utilized for all analyses.

## Results

### Characteristics of population in the observational study

There were 25,911 participants included into the observation study. As shown in Table 1, the population could be grouped into two groups: no sarcopenia (22,211 participants) and sarcopenia (3700 participants). Baseline data, including age, BMI value, gender, race, total energy intake, smoking status and alcohol drinking, showed significant difference between these two groups. Left arm lean mass, left leg lean mass, and appendicular lean mass were significantly higher in no sarcopenia group compared with sarcopenia group. Sarcopenia participants presented a higher population proportion of cancer, 8.757%, compared with no sarcopenia participants, 6.204%.

### Association between sarcopenia relevant traits and cancer

**Figure 1.**
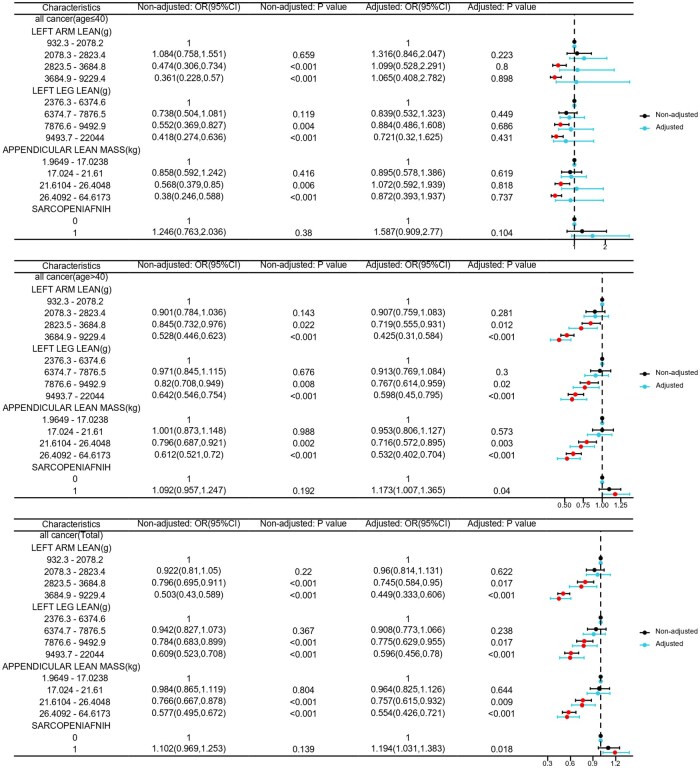
The Multiple regressions results of sarcopenia relevant traits, including left arm lean, left leg lean, appendicular lean mass, and sarcopenia (FNIH) on all cancers in the general USA population. (The analysis was adjusted by gender, race Hispanic origin, BMI value, total energy intake, smoking status and alcohol drinking, and grouped by age (40 years old)).

**Figure 2.**
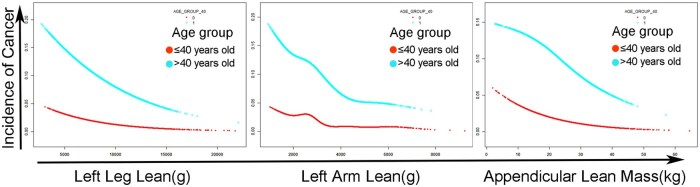
The non-linear association between sarcopenia relevant traits, including left arm lean, left leg lean, appendicular lean mass, and all cancers in the general USA population, analyzed by the smooth curve fitting method. (The analysis was adjusted by gender, race Hispanic origin, BMI value, total energy intake, smoking status and alcohol drinking, and grouped by age (40 years old)).

We analyzed the association between sarcopenia and cancer by four quantile regression models, without or with adjusted covariates. Considering the influence of age on cancer occurrence, we divided population into age ≤ 40 group and age > 40 group. Gender, BMI, race, total energy intake, smoking status, and alcohol drinking were adjusted. As shown in Figure 1, in age ≤ 40 group, sarcopenia relevant traits showed no significant influence on cancer after adjusting covariates. In age > 40 group, left arm lean, left leg lean and appendicular lean mass all showed negative effects on the occurrence of cancer. Significant associations between muscle mass and cancer could be seen in the third subgroup (left arm lean: adjusted OR = 0.719, adjusted P = 0.012; left leg lean: adjusted OR = 0.767, adjusted P = 0.02; appendicular lean mass: adjusted OR = 0.716, adjusted P = 0.003) and fourth subgroup (left arm lean: adjusted OR = 0.425, adjusted P < 0.001; left leg lean: adjusted OR = 0.598, adjusted P < 0.001; appendicular lean mass: adjusted OR = 0.532, adjusted P < 0.001) of these three sarcopenia relevant traits. Sarcopenia (FNIH) could significantly promote the occurrence of cancer in patient at age > 40, with an adjusted OR = 1.173, adjusted P = 0.04. In the total population, left arm lean, left leg lean and appendicular lean mass also showed significantly negative effects on cancer in the third subgroup (left arm lean: adjusted OR = 0.745, adjusted P = 0.017; left leg lean: adjusted OR = 0.775, adjusted P = 0.017; appendicular lean mass: adjusted OR = 0.757, adjusted P = 0.009) and fourth subgroup (left arm lean: adjusted OR = 0.449, adjusted P < 0.001; left leg lean: adjusted OR = 0.596, adjusted P < 0.001; appendicular lean mass: adjusted OR = 0.554, adjusted P < 0.001). Sarcopenia (FNIH criteria) significantly promoted cancer occurrence in the total population (adjusted OR = 1.194, adjusted P = 0.018). Four quantile regression models also showed positive effects of sarcopenia (FNIH) on breast cancer in patients aged ≤ 40(Supplemental Figure S1), there were no significant influence of sarcopenia relevant traits on cervical cancer (Supplemental Figure S2). It identified negative effects of left arm lean mass on colon cancer and positive effects of sarcopenia (FNIH) on colon cancer in patients age > 40 and total population (Supplemental Figure S3), positive effects of left leg lean mass (second subgroup) on melanoma in patients age > 40 (Supplemental Figure S4), and positive effects of sarcopenia (FNIH) on ovarian cancer in patients age > 40 and total population (Supplemental Figure S5). There was no significant influence of sarcopenia relevant traits on skin no melanoma cancer (Supplemental Figure S6). It indicated negative effects of sarcopenia (FNIH) on thyroid cancer in age > 40 group (Supplemental Figure S7), and positive effects of sarcopenia (FNIH) on uterine cancer in age > 40 group and total population (Supplemental Figure S8). The partial results were not consistent with the results found in all cancer population. It could result from the limited number of participants in different cancer diseases grouped by age, as presented in Supplemental Dataset 7. Regression models were also not applicable in some cancer types because of the limited participant number. Besides, the smooth curve fitting method showed the nonlinear correlation between sarcopenia traits and cancer. Figure 2 showed that with the increasement of left arm lean, left leg lean and appendicular lean mass, incidence of cancer gradually decreased, both in age ≤ 40 group and age > 40 group. The analysis was adjusted by gender, race Hispanic origin, BMI value, total energy intake, smoking status and alcohol drinking.

### MR analysis of sarcopenia and cancer

**Figure 3.**
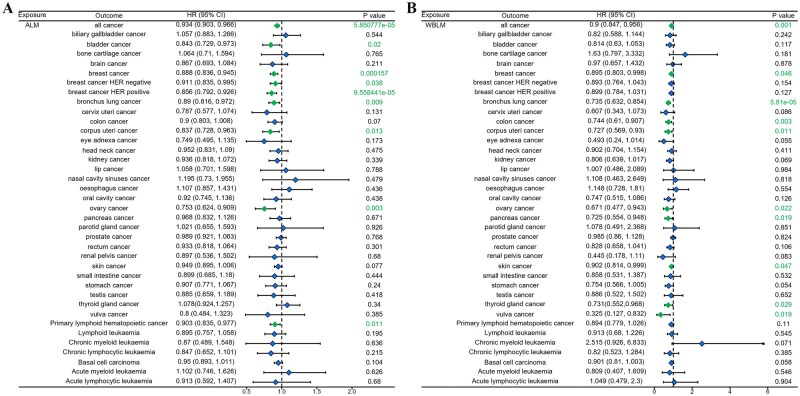
The casual effect of (A) appendicular lean mass and (B) whole body lean mass on cancers analyzed using the IVW method.

**Figure 4.**
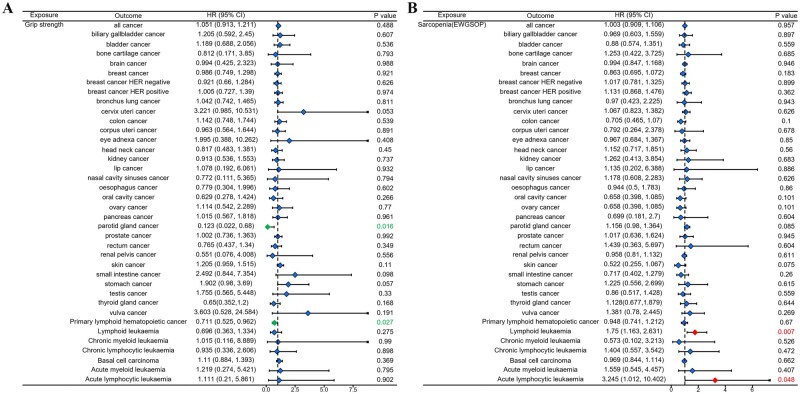
The casual effect of (A) grip strength and (B) sarcopenia (EWGSOP) on cancers analyzed using the IVW method. (EWGSOP: European Working Group on Sarcopenia in Older People).

**Figure 5.**
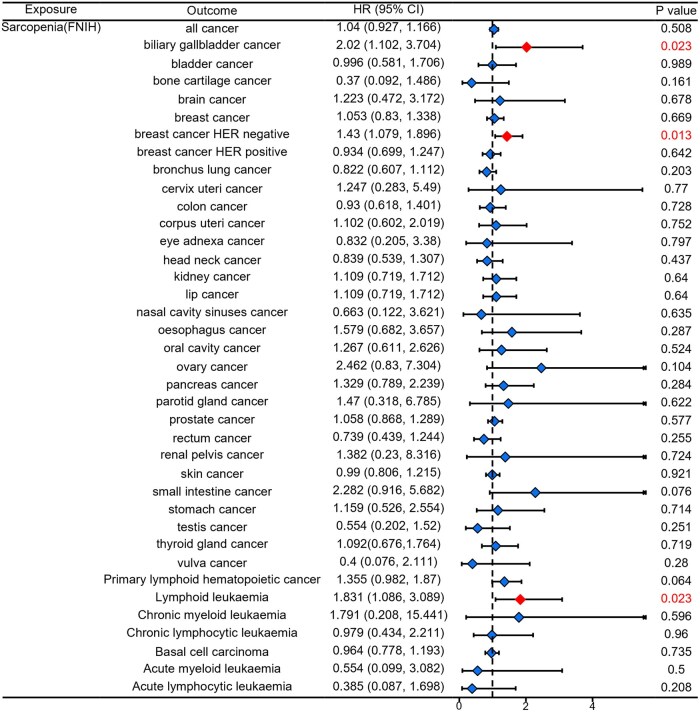
The casual effect of sarcopenia (FNIH) on cancers analyzed using the IVW method. (FNIH: Foundation for the National Institutes of Health).

Then we used MR analysis to investigate whether sarcopenia causally influenced cancer. The results were shown in Figures 3 to 5. All significant findings are summarized in Table 2. The Figure 3A showed that ALM negatively influenced the occurrence of all cancer. ALM causally downregulated the incidence of bladder cancer, breast cancer (all, HER negative and HER positive), bronchus lung cancer, corpus uteri cancer, ovary cancer, and primary lymphoid hematopoietic cancer. The Figure 3B showed that WBLM had causally negative effects on all cancer, breast cancer, bronchus lung cancer, colon cancer, corpus uteri cancer, ovary cancer, pancreas cancer, skin cancer, thyroid gland cancer, and vulva cancer. The Figure 4A showed that grip strength presented causally negative effects on parotid gland caner and primary lymphoid hematopoietic cancer. The Figure 4B showed that sarcopenia (EWGSOP) causally promoted the occurrence of lymphoid leukemia and acute lymphoid leukemia. The Figure 5 showed that sarcopenia (FNIH) causally promoted the occurrence of biliary gallbladder cancer, breast cancer HER negative and lymphoid leukemia, although no significant influence of sarcopenia on all cancer occurrence was observed.

**Table 2.** Summary of significant causal associations from MR analysis of sarcopenia-related traits on various cancers.

Exposure	Outcome	HR (95% CI)	P value
**ALM**	all cancer	0.934 (0.903, 0.966)	5.850777e-05
	bladder cancer	0.843 (0.729, 0.973)	0.02
	breast cancer	0.888 (0.836, 0.945)	0.000157
	breast cancer HER negative	0.911 (0.835, 0.995)	0.038
	breast cancer HER postive	0.856 (0.792, 0.926)	9.558441e-05
	bronchus lung cancer	0.89 (0.816, 0.972)	0.009
	corpus uteri cancer	0.837 (0.728, 0.963)	0.013
	ovary cancer	0.753 (0.624, 0.909)	0.003
	Primary lymphoid hematopoietic cancer	0.903 (0.835, 0.977)	0.011
**WBLM**	all cancer	0.9 (0.847, 0.956)	0.001
	breast cancer	0.895 (0.803, 0.998)	0.046
	bronchus lung cancer	0.735(0.632, 0.854)	5.81e-05
	colon cancer	0.744 (0.61, 0.907)	0.003
	corpus uteri cancer	0.727 (0 569, 0.93)	0.011
	ovary cancer	0.671 (0.477, 0.943)	0.022
	pancreas cancer	0.725 (0.554, 0.948)	0.019
	skin cancer	0.902 (0.814, 0.999)	0.047
	thyroid gland cancer	0.731 (0.552, 0.968)	0.029
	vulva cancer	0.325 (0.127, 0.832)	0.019
**Grip strength (Kg)**	parotid gland cancer	0.123 (0.022, 0.68)	0.016
	Primary lymphoid hematopoietic cancer	0.711 (0.525, 0.962)	0.027
**Sarcopenia (EWGSOP)**	Lymphoid leukaemia	1.75 (1.163, 2.631)	0.007
	Acute lymphocytic leukaemia	3.245 (1.012, 10.402)	0.048
**Sarcopenia (FNIH)**	biliary gallbladder cancer	2.02 (1.102, 3.704)	0.023
	breast cancer HER negative	1.43 (1.079, 1.896)	0.013
	Lymphoid leukaemia	1.831 (1.086, 3.089)	0.023

## Discussion

In this study, we utilized quantile regression (across four quantiles) and MR analyses to investigate the association between sarcopenia and various cancer types. Four quantile regression analyses based on NHANES revealed that left arm lean mass, left leg lean mass and ALM were significantly and inversely associated with overall cancer incidence across all age groups, including individuals under and over 40 years. Further analyses identified strong associations between sarcopenia-related indicators and specific cancers, such as breast, cervical, colon, ovarian, and uterine cancers. The smooth curve also demonstrated a negative correlation between overall cancer incidence and lean mass.

Grip strength as one of the gold standards for diagnosing sarcopenia, peaks around the age of 25 for both genders, with a significant decline after age 40. Therefore, the post-40 period may represent a crucial preclinical phase for sarcopenia onset.^13^ Thus, our quantile regression analysis stratified the data at age 40, revealing significant inverse associations between lean mass and overall cancer incidence for both age groups, but with markedly larger regression coefficients in the over-40 population. It indicated that the pre-sarcopenia period after 40 is critical for preventing sarcopenia and related diseases such as cancer. Interventions during this period may yield significant preventive benefits.

It is recognized that the cross-sectional nature of NHANES data may be prone to confounding bias. Undiagnosed or subclinical cancer may lead to muscle wasting prior to clinical diagnosis, contributing to the observed inverse association between muscle mass and cancer risk. This limitation highlights the value of integrating causal inference methods like MR with observational data to strengthen our conclusions. In this study, MR analysis found that sarcopenia relevant traits, including ALM, WBLM, grip strength, sarcopenia (EWGSOP criteria) and sarcopenia (FNIH criteria), had significantly causal effects on overall cancer and different cancer types. Both ALM and WBLM causally promoted cancer occurrence, with WBLM influencing more cancer types than other traits, indicating that sarcopenia causally promotes cancer.

Sarcopenia is diagnosed through assessments of muscle mass and function, typically identified by low handgrip strength and confirmed by DXA measurements of ALM and WBLM. The EWGSOP and FNIH Sarcopenia Project are commonly used diagnostic criteria.^14^^,^^15^ According to the EWGSOP criteria, males with grip strength below 30 kg and females below 20 kg are classified as having sarcopenia, while the FNIH criteria define sarcopenia as grip strength below 26 kg for males and below 16 kg for females. The EWGSOP criteria aim to identify early-stage sarcopenia, whereas the FNIH criteria define more advanced cases associated with adverse outcomes. Thus, significant associations observed only under the FNIH criteria may suggest a link to more advanced sarcopenia, characterized by substantial muscle loss. Conversely, associations under both criteria may indicate a dose-response relationship, enhancing the credibility of a causal link, as seen with lymphoid leukemia.

Previous studies indicated that patients with cancer tend to develop sarcopenia, leading to poor prognosis and survival outcomes. Compared to patients without cancer, patients with cancer showed a more rapid decline in gait speed before diagnosis and in ALM after diagnosis, with slow gait speed associated with higher mortality risks among patients with cancer.^6^ Sarcopenia may also exacerbate treatment-related toxicity in patients undergoing chemotherapy. A population-based study revealed that reduced fat-free mass contributes to increased chemotherapy toxicity in sarcopenic obesity cases.^16^ Additionally, interventions targeting sarcopenia may benefit patients with cancer. For instance, exercise has been shown to alleviate cancer-related fatigue and enhance physical function.^17^ Given that muscle loss is often attributed to chronic inflammation, anti-inflammatory medications have also been explored to mitigate weight loss in patients with cancer.^18^ While these studies emphasize the importance of preventing and treating sarcopenia to improve cancer prognosis, few have explicitly examined whether individuals with sarcopenia are at higher risk of developing cancer.

Our results discovered a causal relationship between sarcopenia and cancer incidence, with sarcopenia contributing to overall cancer incidence and promoting various cancers. For urologic cancer, bladder cancer significantly increased sarcopenia incidence, indicating an interaction that worsens cancer progression.^19^ It found that increased muscle mass had causally protective effects against bladder cancer from a genetic perspective, suggesting that improving muscle mass may benefit bladder cancer prevention. For respiratory cancer, our MR analysis indicated genetically causal effects of sarcopenia on bronchus lung cancer. The prevalence of sarcopenia in patients with lung cancer was reported to be 42.8% to 45%, and sarcopenia correlates with increased postoperative complications and reduced efficacy of immune checkpoint inhibitors.^20^ For gynecological cancers, our findings suggested potential risks of sarcopenia on vulvar cancer, though limited studies have reported this effect. The relationship between ovarian cancer and sarcopenia remains unclear, with some studies not observing significant impacts on survival and complications.^21^^,^^22^ However, both MR and quantile regression analyses in our study supported a risk-increasing effect of sarcopenia on ovarian cancer. Therefore, more prospective clinical research is needed to clarify the relationship. Sarcopenia had a prevalence of 45% to 50% in patients with breast cancer.^23^^,^^24^ Our results confirmed the protective effects of muscle mass on breast cancer, along with the risk-increasing effect of sarcopenia in individuals under age 40. The MR analyses demonstrated that both ALM and WBLM exhibited significant negative causal effects on the overall incidence of breast cancer. Notably, the lower HR and more significant P-value indicated a more pronounced causal effect for ALM than for WBLM. Furthermore, ALM showed a particularly strong causal association with HER-positive breast cancer subtypes. In the context of sarcopenia definitions, only sarcopenia defined by the more stringent FNIH criteria was significantly causally associated with overall breast cancer incidence, whereas no such association was observed under the EWGSOP criteria. Compared to WBLM, ALM more specifically reflects skeletal muscle mass and is widely adopted as a key diagnostic criterion for sarcopenia. Collectively, these findings indicate that muscle mass, rather than muscle function, may substantially influence breast carcinogenesis, potentially through biological mechanisms involving HER2 signaling. For uterine cancer, including cervical cancer, the presence of sarcopenia correlated with worse outcomes, especially in sarcopenic obesity cases. Patients with uterine cancer also exhibited higher sarcopenia incidence.^25^^,^^26^ Our MR analyses observed protective effects of muscle mass on uterine cancer and demonstrated that muscle mass causally influenced this cancer type.

For digestive system cancers, we identified causally risky effects of sarcopenia on colon cancer, supported by observational analysis. A retrospective study noted higher secondary cancer incidence in sarcopenic patients post-curative resection of rectal cancer, indicating that sarcopenia possibly contributed to the development of colorectal cancer.^27^ We also found a causal association between sarcopenia and increased biliary gallbladder cancer risk, while higher muscle mass exhibited protective effects against pancreatic cancer. Although previous studies correlated sarcopenia with poorer outcomes in biliary tract and pancreatic cancers,^28^^,^^29^ our study is the first to elucidate, from a genetic perspective, the causal role of sarcopenia in these malignancies. In NHANES analysis, sarcopenia did not significantly associate with non-melanoma skin cancer, yet MR analysis demonstrated significant causal effects of muscle mass on skin cancer risk, consistent with research indicating sarcopenia adversely affects outcomes in metastatic melanoma.^30^ Limited studies have reported on the association between sarcopenia and parotid gland cancer, though some studies suggest correlations with poorer outcomes in malignant salivary gland tumors.^31^ Hematologic malignancies, particularly lymphocytic leukemia, were identified as causally influenced by sarcopenia or reduced muscle mass, suggesting that improving muscle mass and treating sarcopenia may enhance survival and quality of life for patients with cancer. Previous studies linked sarcopenia to inferior survival in chronic lymphocytic leukemia patients receiving novel therapies.^32^ ­Conflicting results were observed between the MR and NHANES-based analyses regarding the association of sarcopenia with thyroid cancer. possibly due to limited participants in NHANES for thyroid cancer analysis. Current literature indicates sarcopenia correlates with worse survival in patients with advanced thyroid cancer.^33^ Therefore, these observational findings warrant further validation through larger prospective clinical studies.

The potential mechanisms by which sarcopenia influences cancer development and progression are multifaceted. Firstly, it is linked to chronic inflammation in the muscles, resulting in increased pro-inflammatory cytokines like IL-6 and TNF-α, which promote tumor cell proliferation and invasion while inhibiting apoptosis through pathways such as STAT3/NF-κB.^34–36^ This inflammatory environment can also expand myeloid-derived suppressor cells (MDSCs) and suppress ­cytotoxic T cell function, weakening anti-tumor immune responses.^37^ Secondly, skeletal muscle, the largest tissue in the body, plays a vital role in systemic metabolic regulation. Muscle atrophy disrupts metabolic homeostasis, creating conditions favorable for tumor growth. Significant muscle loss can lead to dysregulated amino acid metabolism and increased lipolysis, resulting in hyperlipidemia and insulin resistance, which tumor cells can exploit for rapid proliferation.^38^^,^^39^ Finally, muscle atrophy affects myokine regulation, with irisin potentially inhibiting tumor growth.^40^ Muscle mass is closely related to hormones such as estrogen, thyroid hormones, and cortisol, and dysregulation of these can create an endocrine environment that promotes tumor development.^41^ Emerging research also suggests that skeletal muscle acts as an endocrine organ, influencing other organs through paracrine or endocrine pathways.^42^ Overall, while further investigation is needed, our study offers genetic evidence supporting sarcopenia’s causal role in the development and progression of various cancer types, highlighting the clinical importance of sarcopenia management in oncology care.

Compared to previous studies, our research presents notable methodological strengths. This is the first study combining quantile logistic regression of NHANES data with MR analysis of large-scale GWAS summary statistics to examine sarcopenia’s causal role in diverse cancers. The MR approach minimizes reverse causation and confounding issues, while quantile regression enables detailed outcome association examination. This combined framework enhances the robustness of our causal inferences. However, limitations exist, including a relatively limited number of cancer cases in the NHANES cohort, potentially affecting statistical power. We triangulated evidence from MR and NHANES to bolster inference robustness. Additionally, while this study focused on etiological role of sarcopenia in cancer development, it did not address its impact on cancer survival or patient-centered outcomes. Future studies should investigate influence of sarcopenia on prognosis and the potential of targeted interventions to improve life quality of patients with cancer. Finally, as both GWAS and NHANES datasets primarily include individuals of European and U.S. ancestry, the findings may have limited generalizability to other ethnic groups. Further validation in multi-ethnic cohorts is necessary to confirm the broader relevance of our conclusions.

## Conclusion

In conclusion, this study provides robust evidence supporting a causal relationship between sarcopenia-related traits and an increased risk of multiple cancer types, through an observational study of the NHANES dataset combined with Mendelian randomization analysis of GWAS data. Our findings highlight the importance of maintaining muscle mass, particularly as measured by ALM in cancer prevention strategies, especially for HER2-positive breast cancer and lymphoid leukemia. These results underscore the potential significance of muscle mass in cancer development. However, these findings require validation in multi-ethnic cohorts to ensure broader applicability beyond populations of European ancestry. Future research should focus on elucidating the underlying biological mechanisms and exploring whether interventions targeting sarcopenia could reduce cancer incidence or improve outcomes in patients with cancer.

## Supplementary Material

oyaf369_Supplementary_Data

## Data Availability

The NHANES based analysis was approved by National Center for Health Statistics (NCHS) Ethics Review Board (ERB) with protocol numbers as #98-12, #2005-06, #2011-17 and #2018-01. All participants have assigned the informed consent forms. The data used in this study are available on the NHANES website (https://www.cdc.gov/nchs/nhanes/index.htm). All GWAS data were obtained with informed consent from participants in the originating institutions. Therefore, these publicly available data could use in this study without requiring further ethical approvement. The data that support the findings of this study are available from the corresponding author upon reasonable request.
