# Supplementary material for: Contributing effects of sarcopenia on cancer occurrence: novel evidence based on NHANES 1999–2020 and two-sample mendelian randomization study
Source: Oncologist. 2025 Nov 5;30(11):oyaf369. doi: 10.1093/oncolo/oyaf369 (PMC12628308; doi:10.1093/oncolo/oyaf369)
Supplement: oyaf369_Supplementary_Data [file oyaf369_supplementary_data.zip › supplementary figures S1-S8 and figure legends.docx]

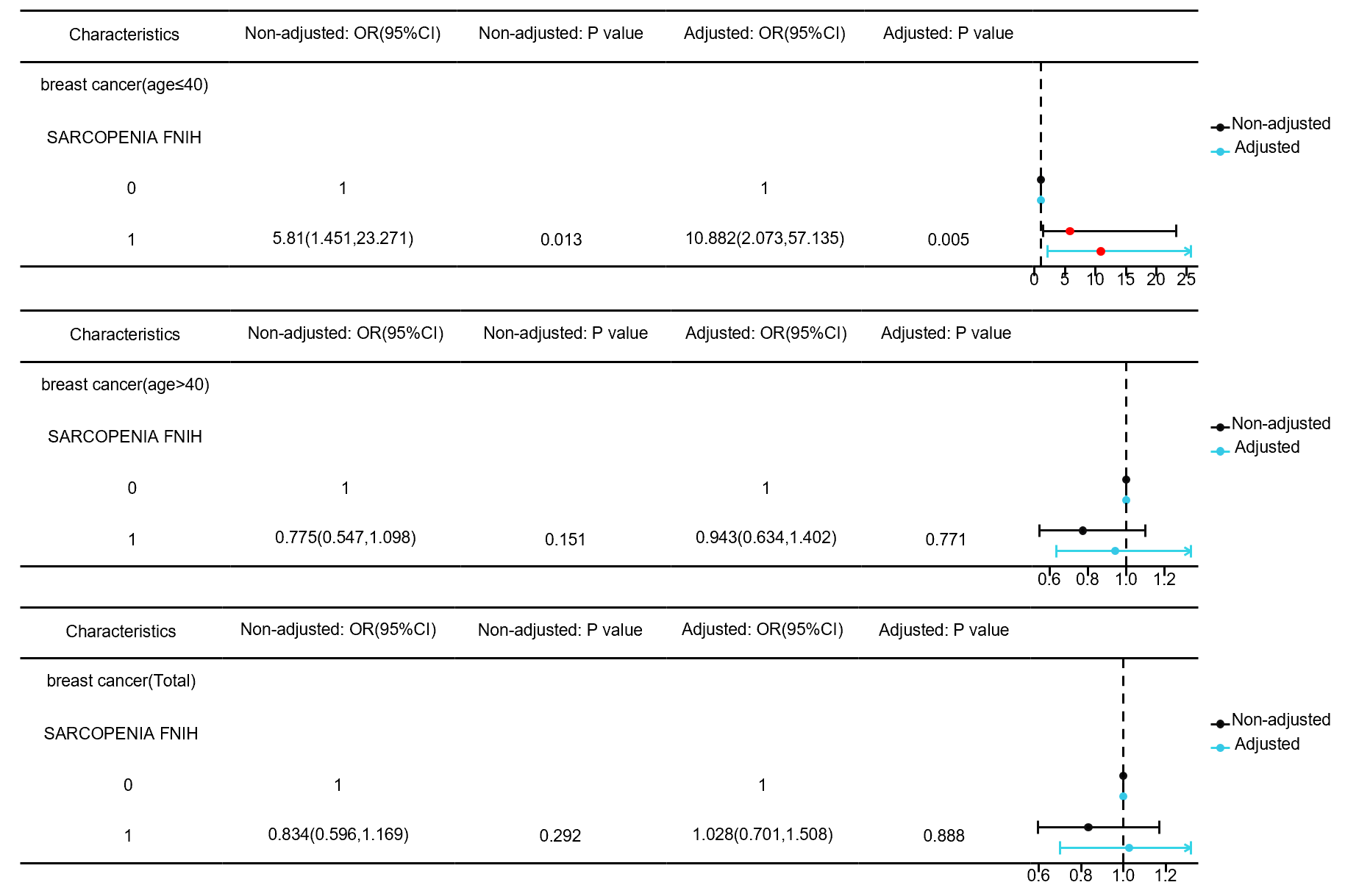


Figure S1. The Multiple regressions result of sarcopenia relevant traits, including left arm lean, left leg lean, appendicular lean mass, and sarcopenia (FNIH) on breast cancer in the general USA population. [The analysis was adjusted by gender, race Hispanic origin, BMI value, total energy intake, smoking status and alcohol drinking, and grouped by age (40 years old)].


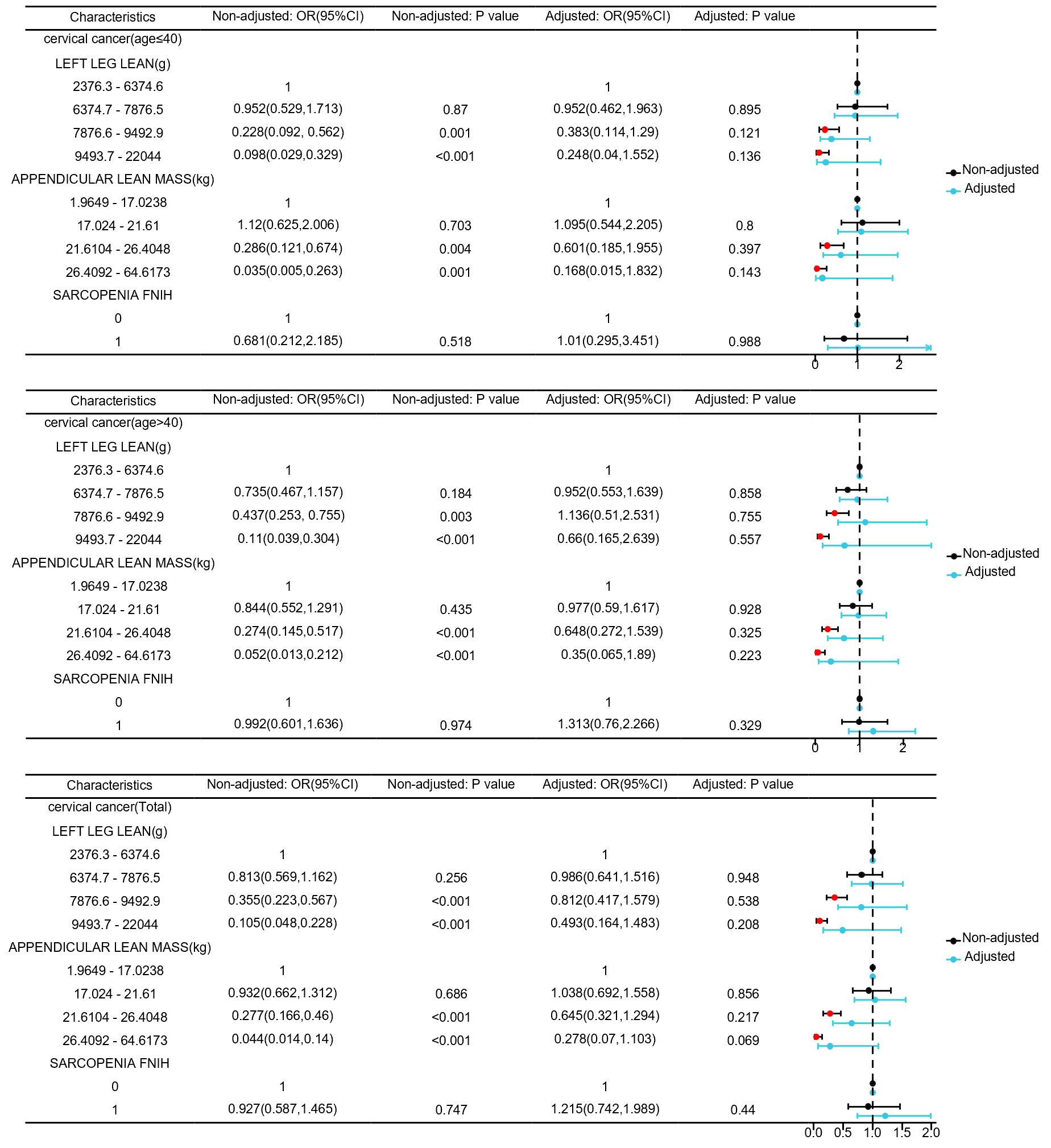


Figure S2. The Multiple regressions result of sarcopenia relevant traits, including left arm lean, left leg lean, appendicular lean mass, and sarcopenia (FNIH) on cervical cancer in the general USA population. [The analysis was adjusted by gender, race Hispanic origin, BMI value, total energy intake, smoking status and alcohol drinking, and grouped by age (40 years old)].


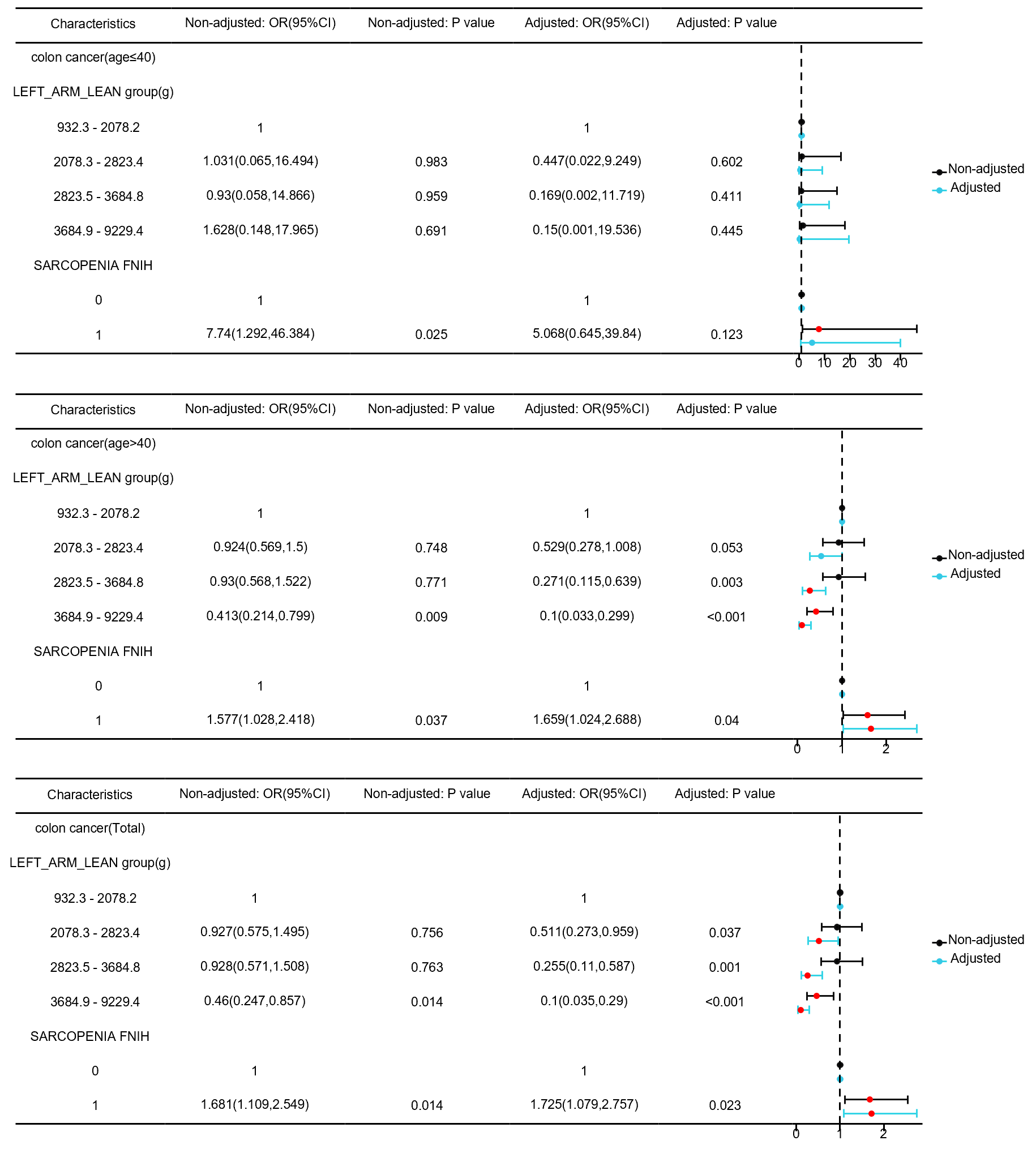


Figure S3. The Multiple regressions result of sarcopenia relevant traits, including left arm lean, left leg lean, appendicular lean mass, and sarcopenia (FNIH) on colon cancer in the general USA population. [The analysis was adjusted by gender, race Hispanic origin, BMI value, total energy intake, smoking status and alcohol drinking, and grouped by age (40 years old)].


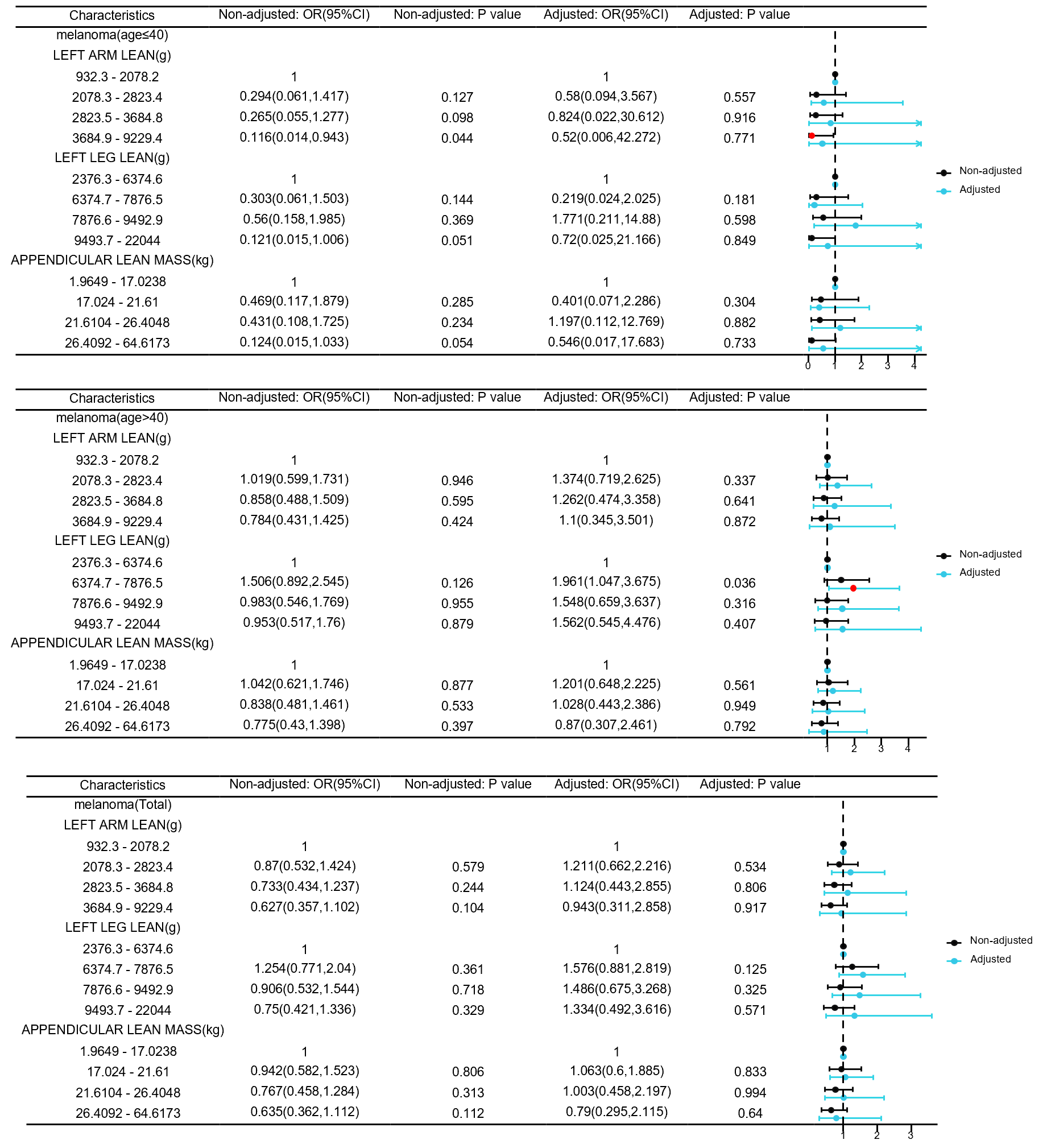


Figure S4. The Multiple regressions result of sarcopenia relevant traits, including left arm lean, left leg lean, appendicular lean mass, and sarcopenia (FNIH) on melanoma in the general USA population. [The analysis was adjusted by gender, race Hispanic origin, BMI value, total energy intake, smoking status and alcohol drinking, and grouped by age (40 years old)].


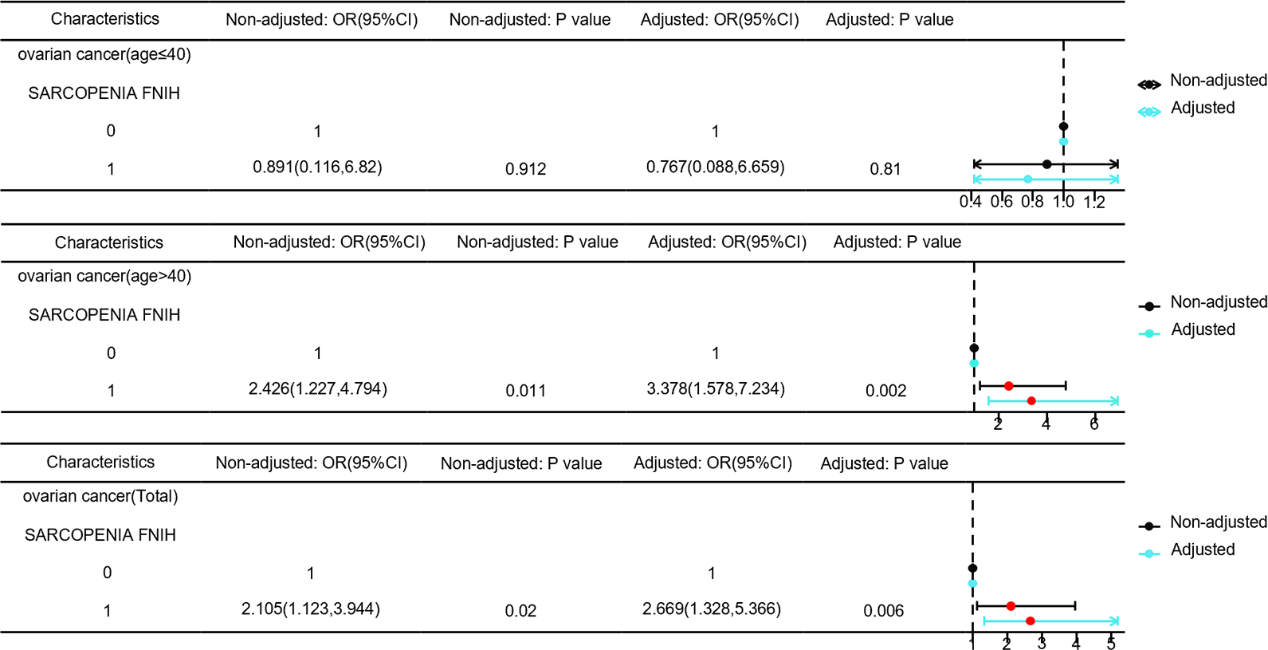


Figure S5. The Multiple regressions result of sarcopenia relevant traits, including left arm lean, left leg lean, appendicular lean mass, and sarcopenia (FNIH) on ovarian cancer in the general USA population. [The analysis was adjusted by gender, race Hispanic origin, BMI value, total energy intake, smoking status and alcohol drinking, and grouped by age (40 years old)].


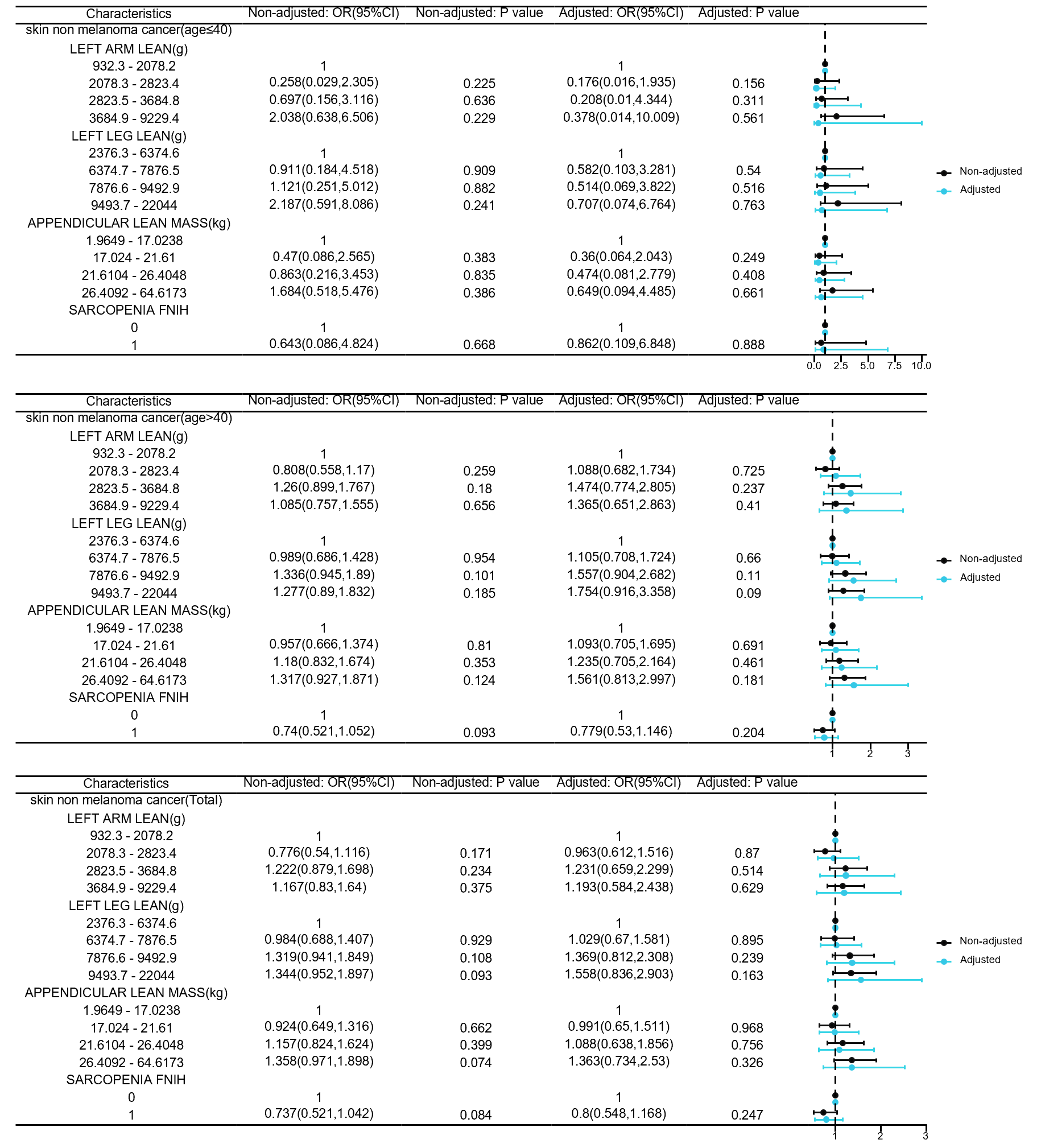


Figure S6. The Multiple regressions result of sarcopenia relevant traits, including left arm lean, left leg lean, appendicular lean mass, and sarcopenia (FNIH) on skin non melanoma cancer in the general USA population. [The analysis was adjusted by gender, race Hispanic origin, BMI value, total energy intake, smoking status and alcohol drinking, and grouped by age (40 years old)].


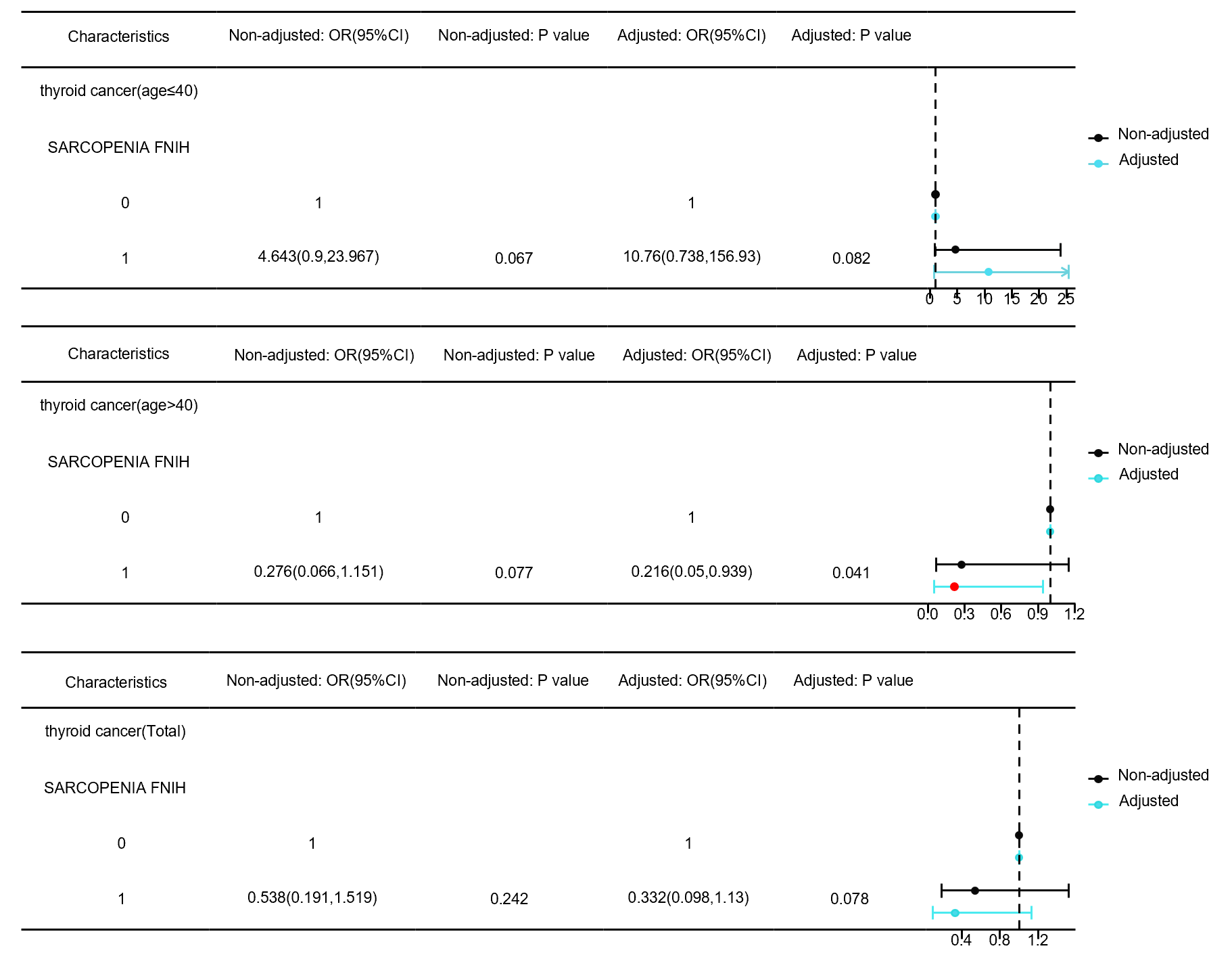


Figure S7. The Multiple regressions result of sarcopenia relevant traits, including left arm lean, left leg lean, appendicular lean mass, and sarcopenia (FNIH) on thyroid cancer in the general USA population. [The analysis was adjusted by gender, race Hispanic origin, BMI value, total energy intake, smoking status and alcohol drinking, and grouped by age (40 years old)].


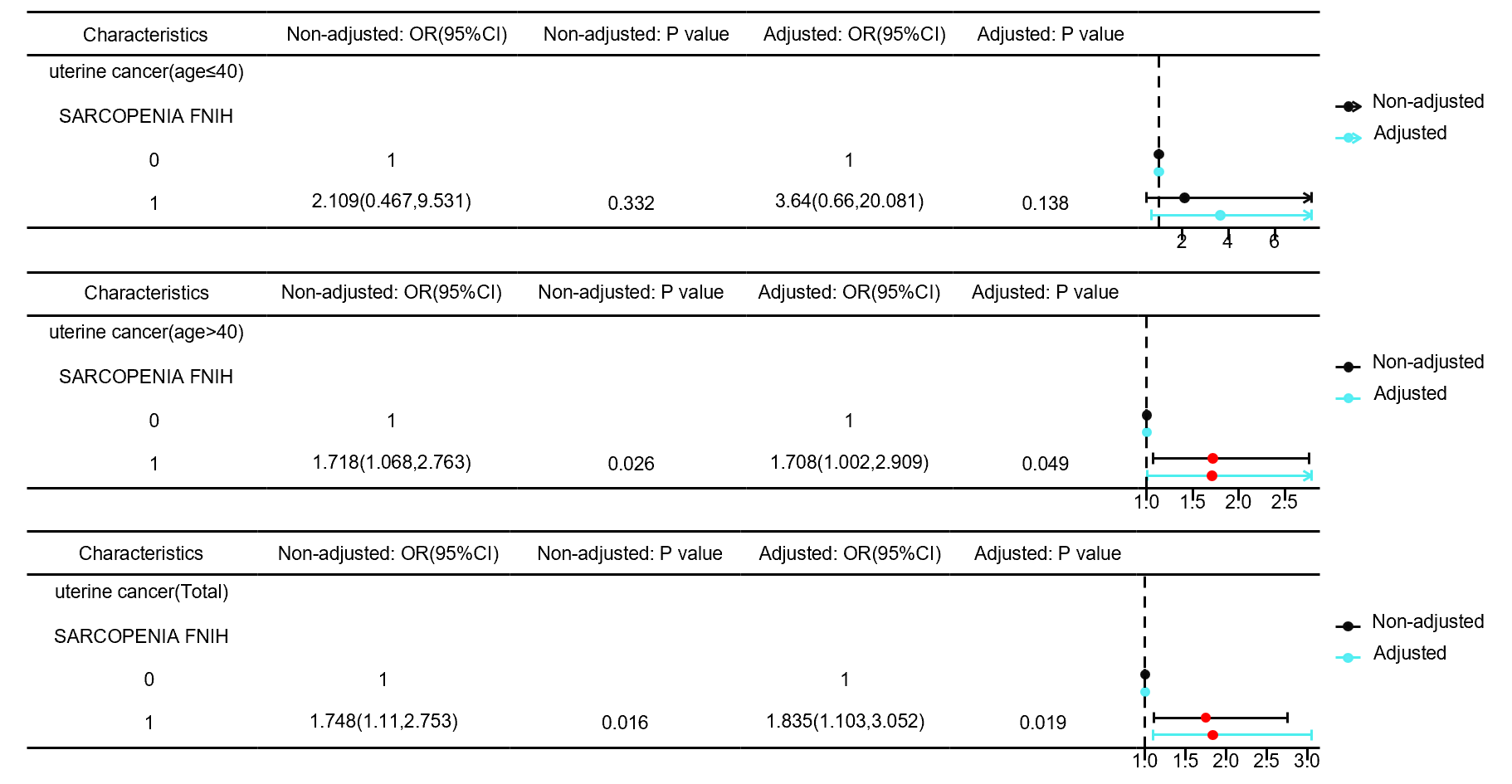


Figure S8. The Multiple regressions result of sarcopenia relevant traits, including left arm lean, left leg lean, appendicular lean mass, and sarcopenia (FNIH) on uterine cancer in the general USA population. [The analysis was adjusted by gender, race Hispanic origin, BMI value, total energy intake, smoking status and alcohol drinking, and grouped by age (40 years old)].
